# Tuberculosis Presenting as Migratory Arthritis: A Case Report from Iraq

**DOI:** 10.1002/ccr3.72238

**Published:** 2026-03-09

**Authors:** Farah Jaafar Mahdi, Mariam Salem Othman, Ahmed Dheyaa Al‐Obaidi, Mustafa Almusawi, Marafi Jammaa Ahmed, Aya Ahmed Shimal, Elian Khalafalla Awadalla, Marwah Algodi

**Affiliations:** ^1^ Rheumatology Unit, Department of Medicine, College of Medicine University of Mustansiriyah Baghdad Iraq; ^2^ Faculty of Medicine Alexandria University Alexandria Egypt; ^3^ College of Medicine University of Baghdad Baghdad Iraq; ^4^ Nerve and Muscle Center of Texas Baylor St. Luke Medical Center Houston Texas USA; ^5^ Faculty of Medicine University of Bahri Khartoum Sudan; ^6^ College of Medicine University of Khartoum Khartoum Sudan; ^7^ Department of Internal Medicine Hackensack Meridian Health – Jersey Shore University Medical Center Neptune City New Jersey USA

**Keywords:** extrapulmonary tuberculosis, migratory arthritis, *Mycobacterium tuberculosis*, Poncet's disease, rheumatoid arthritis mimic, tuberculosis

## Abstract

Extrapulmonary tuberculosis is uncommon, and musculoskeletal involvement presenting as migratory arthritis is particularly rare in immunocompetent individuals, often leading to misdiagnosis and delayed treatment. We report the case of a 66‐year‐old man with diabetes mellitus who presented with recurrent migratory inflammatory arthritis affecting multiple joints over 1 year, without initial pulmonary symptoms. He was initially diagnosed with rheumatoid arthritis based on positive rheumatoid factor and elevated inflammatory markers and was treated with disease‐modifying antirheumatic drugs without clinical improvement. At presentation, he reported severe shoulder pain, morning stiffness, weight loss, and night sweats. Imaging revealed right upper lobe consolidation and a metabolically active cavitary lung lesion on PET‐CT. Bronchoalveolar lavage culture confirmed *Mycobacterium tuberculosis* . Antituberculous therapy was initiated, resulting in marked clinical improvement and resolution of arthritis within weeks. This case highlights tuberculosis as an important, underrecognized cause of migratory arthritis and emphasizes the need to consider infectious etiologies in treatment‐resistant inflammatory joint disease, particularly in endemic regions.

## Key Clinical Message

Tuberculosis should be considered in patients with treatment‐resistant inflammatory arthritis or rheumatoid arthritis–like presentations, particularly when constitutional symptoms are present or in endemic settings. Infectious etiologies must be excluded before initiating or escalating immunosuppressive therapy. Early recognition of atypical extrapulmonary tuberculosis can prevent misdiagnosis, inappropriate immunosuppression, and delay in definitive treatment.

## 1 Introduction

Tuberculosis (TB), caused by *Mycobacterium tuberculosis* , remains a major global health concern, particularly in developing countries. While pulmonary TB accounts for approximately 85% of cases, extrapulmonary TB (EPTB) represents about 15% in HIV‐negative individuals. EPTB can involve nearly any organ system, most commonly the lymph nodes, pleura, bones, and meninges [1]. Musculoskeletal TB constitutes 10% of all EPTB cases worldwide, with spinal TB representing 50% of cases, followed by cases in the hips and knees [2].

The clinical presentation of active TB varies widely, ranging from asymptomatic cases to severe illness. However, when symptoms are present, they are often nonspecific, posing a significant diagnostic challenge [3]. This is especially true for EPTB, which can mimic other diseases depending on the site involved. Tuberculous arthritis, known as Poncet's disease, may resemble autoimmune joint conditions such as rheumatoid arthritis (RA). The differential diagnosis of migratory arthritis is broad and includes infectious causes, autoimmune diseases, degenerative diseases, crystal‐induced arthropathies, and vasculitic syndromes [4]. Migratory joint pain is an uncommon manifestation of TB and may lead to misdiagnosis or delayed treatment. Therefore, a detailed history, physical examination, and appropriate laboratory investigations are essential for accurate diagnosis.

We present a case of pulmonary TB manifesting as migratory arthritis in an immunocompetent patient, initially misdiagnosed as RA. This case underscores the importance of considering TB in the differential diagnosis of unexplained, treatment‐resistant arthritis, particularly in endemic areas or when systemic signs are present despite the absence of pulmonary symptoms.

## 2 Case History/Examination

A 66‐year‐old male with a 10‐year history of diabetes mellitus, controlled on oral treatment, complained of recurrent attacks of migratory joint pain and swelling that recurred every 2 weeks in a different joint, starting 1 year before presentation. Initially, symptoms began in the right wrist and lasted for a few days before resolving spontaneously. Subsequently, the pain and swelling migrated to the right knee, followed by the left hip, prompting him to seek medical advice. A mistaken diagnosis of RA resulted from investigations that showed a positive rheumatoid factor and an elevated erythrocyte sedimentation rate (ESR). The patient did not show any improvement even after taking methotrexate and hydroxychloroquine. At first, the patient was given 15 mg of methotrexate once a week. The treatment was changed to leflunomide 20 mg daily and then to hydroxychloroquine 200 mg daily due to the insufficient response. He underwent several sessions of high‐dose systemic corticosteroids during flare‐ups of the condition.

The patient is married with four children and resides with his family in an urban, non‐crowded house. The patient does not smoke or consume alcohol. There was no recent travel history, exposure to animals (including birds), or known contact with TB. He had no drug allergies, no relevant family history of similar illnesses, and no significant past surgical history. His vaccination history was up to date with the Iraqi vaccination schedule, including two doses of the Pfizer‐BioNTech COVID‐19 vaccine.

Upon presentation to our rheumatology outpatient clinic, his chief complaint was constant right shoulder pain, which he described as gradual in onset and progressively worsening to a severity of 9/10 on the patient's visual analogue scale (VAS). The pain was dull, aggravated by sleep, and associated with morning stiffness lasting more than 30 min. He expressed that this pain impaired his ability to carry out daily activities. Additionally, he reported low‐grade fever (unmeasured) and night sweats (not drenching). The patient had experienced unintentional weight loss of approximately 15 kg over the previous 8 months, along with fatigue. He denied chills, rigours, cold or heat intolerance, and excessive thirst.

On examination, the patient was conscious and oriented to time and place but appeared pale, cachectic, and in distress. Vital signs were within normal limits, and oxygen saturation was 94% on room air. There were no skin abnormalities and no mucocutaneous ulcers. There was no jaundice or cyanosis. Oral hygiene was good, with normal buccal mucosa and no aphthous ulcers, gingival bleeding, or hypertrophy. Cardiovascular examination revealed no jugular venous distension (JVD), carotid bruits, palpable masses or cervical lymphadenopathy.

Upper limb examination showed pallor of the palmar creases and finger clubbing, characterized by loss of the Lovibond angle and increased curvature of the nail bed (Stage 2 clubbing). Clubbing was thought to be related to chronic pulmonary changes, although no prior lung disease had been diagnosed. There was no peripheral cyanosis, koilonychias, tar staining, scars, rash, or epitrochlear or axillary lymphadenopathy. Shoulder examination demonstrated a tense swelling with mild erythema of the overlying skin. The joint was warm and tender to the touch with a limited range of motion in all directions.

Chest inspection revealed a symmetrical and normally shaped thorax with no deformities, dilated veins or scars. Palpation showed a centrally located trachea, symmetrical chest expansion and increased tactile fremitus in the right upper zone. Percussion elicited dullness in the right upper zone with resonance elsewhere. Auscultation revealed vesicular breathing of normal intensity all over the chest except in the right upper zone, where bronchial breathing with fine inspiratory crackles, positive egophony, and whispering pectoriloquy were noted. Increased vocal resonance was also present in this area and was normal elsewhere. Examination of the remaining systems, including the genitourinary, gastrointestinal, and nervous system, was unremarkable.

## 3 Differential Diagnosis, Investigations, and Treatment

At initial presentation, RA was suspected due to migratory joint symptoms, positive rheumatoid factor, and elevated inflammatory markers. However, the diagnosis was reconsidered due to the absence of a clinical response to DMARDs. Systemic symptoms that raised questions about other differentials including infection or malignancy, included weight loss, low‐grade fever, and night sweats. Further imaging showed lung consolidation, and a cavitary lesion shifted the diagnosis to an infectious disease. The main suspect was TB, which BAL culture later confirmed. The results of laboratory tests revealed a slightly higher level of anti‐CCP antibody at 35 U/mL (normal < 30), an erythrocyte sedimentation rate (ESR) of 102 mm/h (normal < 20), and a C‐reactive protein (CRP) of 46 mg/L (reference < 10 mg/L). A complete blood count showed neutrophil predominance and moderate leukocytosis (WBC 10.5 × 10^9^/L). The findings of the liver and renal function tests fell within normal limits. Features of oxidative stress and low‐grade hemolysis were observed on the peripheral blood smear, which can happen when there is systemic inflammation and a persistent infection.

After thorough counseling, the patient denied the indicated joint aspiration of the right shoulder to rule out septic arthritis, including tuberculous septic arthritis.

**FIGURE 1:**
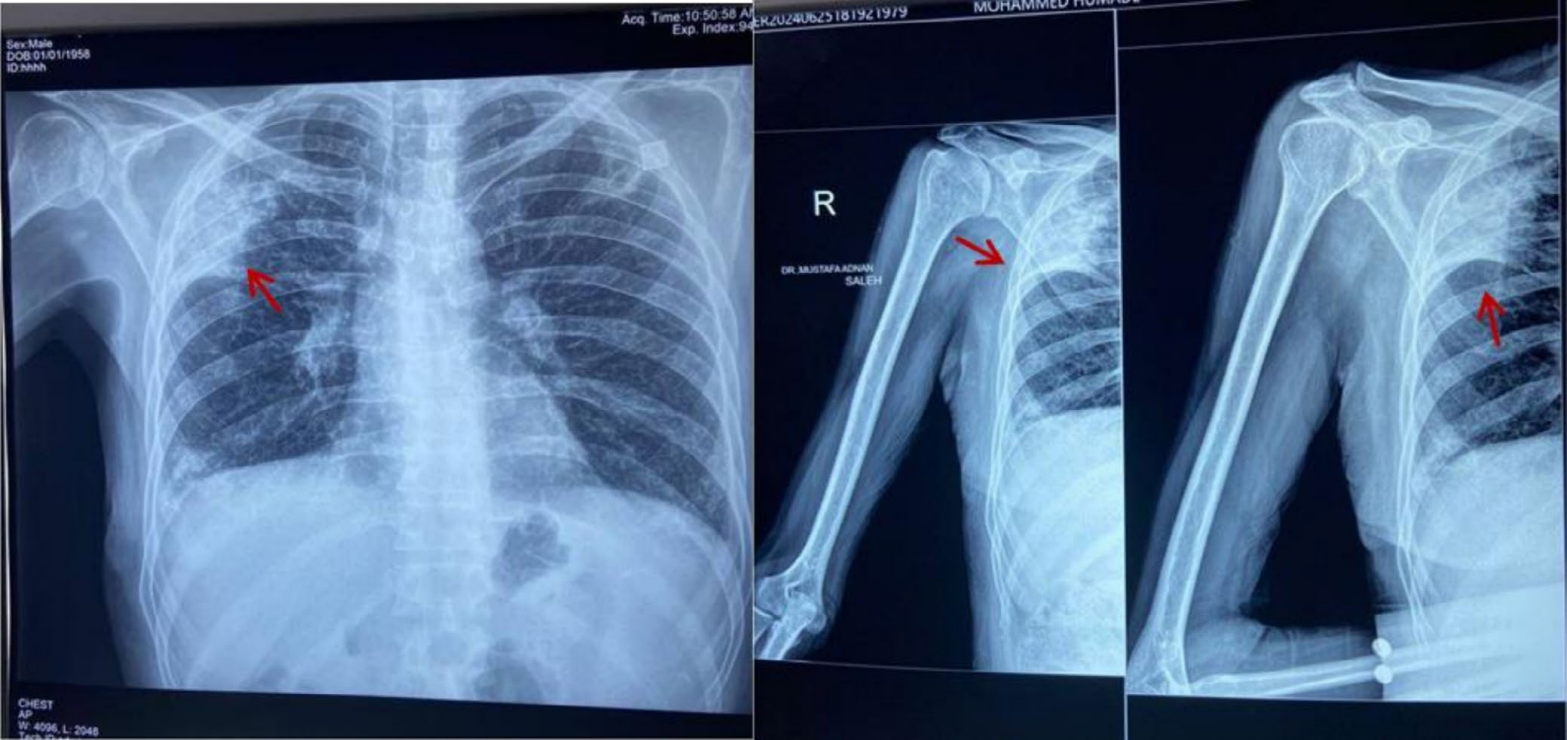
Left: Right shoulder x‐ray showing incidental lung opacity (arrow). Right: Chest x‐ray demonstrating consolidation in the right upper lung lobe (arrow).

**FIGURE 2:**
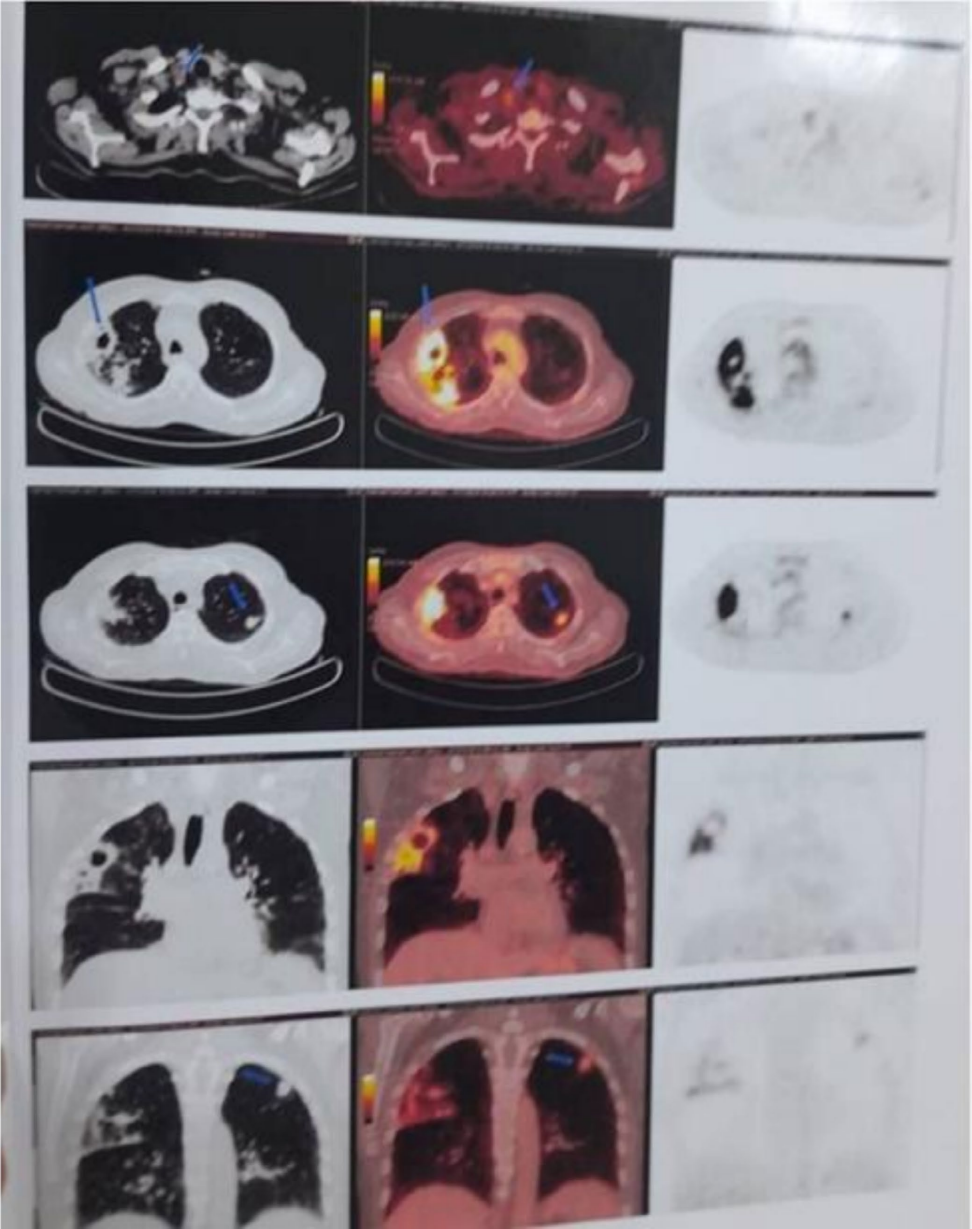
PET CT scan findings are suggestive of a metabolically active cavitary lesion in the right upper lobe, along with bilateral pulmonary nodules. Low‐grade metabolically active right, lower cervical, right mediastinal, and right hilar lymph nodes on the right side were noted. No evidence of any metabolically active disease observed elsewhere.

A right shoulder x‐ray demonstrated degenerative changes of the acromioclavicular joint (AC) with diffuse osteopenia. Incidentally, a lung opacity was noted, prompting further evaluation with a chest x‐ray, which revealed consolidation in the right upper lung lobe (Figure 1). MRI of the right shoulder demonstrated minimal bone marrow edema around the AC joint and joint hypertrophy, suggesting arthropathy. A non‐contrast chest CT scan revealed bronchiectasis and fibrotic alterations with peripheral honeycombing. Since there was no prior diagnosis of interstitial lung illness and the cavitary lesion received clinical care, these were deemed incidental and unrelated to the patient's current TB. There was no documented history of chronic pulmonary illness in the patient. The main finding suggested tuberculosis was the active cavitary lesion in the right upper lobe. Given ongoing concern for malignancy, oncology was consulted, and PET CT imaging was obtained, which demonstrated a metabolically active cavitary lesion along with bilateral pulmonary nodules in the upper lobes (Figure 2). The PET‐avid cervical, mediastinal, and hilar lymph nodes were interpreted as reactive/inflammatory in the context of active pulmonary tuberculosis and were not sampled. These findings shifted clinical suspicion from an autoimmune to an infectious or neoplastic etiology, as hypermetabolic consolidation and cavitary lesions are atypical in RA.

Initially, interferon‐gamma release assay (QuantiFERON‐TB Gold) performed on peripheral blood, acid‐fast bacilli (AFB) smear, and nucleic acid amplification testing (Xpert MTB/RIF) performed on bronchoalveolar lavage (BAL) were negative for tuberculosis. However, mycobacterial culture of the BAL specimen became positive for *Mycobacterium tuberculosis* after 8 weeks of incubation. Drug susceptibility testing did not demonstrate rifampicin resistance. No malignant cells were identified on cytology. For the management timeline, see Table 1.

**TABLE 1:** Chronological timeline of clinical events, investigations, and management.

Time frame	Event
12 months before presentation	Onset of recurrent migratory inflammatory arthritis affecting different joints approximately every 2 weeks
Shortly after symptom onset	Laboratory testing revealed positive rheumatoid factor (RF) and mildly elevated anti‐CCP antibodies; diagnosed with rheumatoid arthritis
Following diagnosis	Methotrexate initiated
Subsequent months	Inadequate clinical response → switched to leflunomide
Later months	Persistent symptoms → switched to hydroxychloroquine; no significant improvement
At presentation to our rheumatology clinic	Severe right shoulder arthritis with constitutional symptoms (weight loss, night sweats, low‐grade fever)
Same visit	Shoulder x‐ray revealed incidental right upper lobe opacity
Within 2 days	Chest CT demonstrated right upper lobe cavitary lesion
Shortly thereafter	PET‐CT showed metabolically active cavitary lesion with pulmonary nodules
Initial recommendation	Bronchoscopy advised; patient declined
Approximately 1 month later	Patient consented to bronchoscopy; bronchoalveolar lavage (BAL) performed
Subsequent result	BAL culture positive for *Mycobacterium tuberculosis*
Immediately after confirmation	Antituberculous therapy initiated
Within 1 month of therapy	Marked clinical improvement and resolution of arthritis

## 4 Outcome and Follow‐Up

A diagnosis of pulmonary tuberculosis with extrapulmonary manifestations presenting as migratory arthritis was confirmed. For a total planned duration of 6 months, the patient was started on standard first‐line antituberculous medication, including isoniazid, rifampicin, pyrazinamide, and ethambutol for 2 months (intense phase), followed by isoniazid and rifampicin for four more months (continuation phase).

The patient showed significant clinical improvement after 4 weeks of starting treatment, with fever and night sweats going away, morning stiffness going away, and joint pain and swelling completely resolved. A follow‐up laboratory analysis revealed that inflammatory indicators had returned to normal, with ESR and CRP falling back within acceptable ranges.

The patient also reported improvement in appetite and stabilization of weight. Radiological follow‐up with repeat chest imaging was planned to assess treatment response.

## 5 Discussion

Migratory arthritis is a diagnosis characterized by transient joint inflammatory activity at various locations sequentially. It is caused by multiple conditions, which include infectious diseases and systemic conditions such as gout, pseudogout, gonococcal infection, familial Mediterranean fever (FMF), and occasionally systemic lupus erythematosus (SLE) or reactive arthritis [3]. TB, while most commonly presenting as pulmonary disease, may also manifest with extrapulmonary involvement, including musculoskeletal presentations such as migratory arthritis [4].

We present a case of an immunocompetent patient with diabetes mellitus who presented with migratory arthritis. Although the patient had low‐titer anti‐CCP antibodies and a positive rheumatoid factor, several clinical features were atypical for rheumatoid arthritis and should have prompted reconsideration of the diagnosis. In particular, RA, which usually manifests as a persistent, symmetric polyarthritis, seldom exhibits the migratory pattern of arthritis, which is defined by temporary involvement of other joints with spontaneous remission. Furthermore, because rheumatoid factor and low‐titer anti‐CCP antibodies can be found in chronic illnesses, such as TB, due to nonspecific immune activation, seropositivity alone is not enough to diagnose RA. In our case, the anti‐CCP increase was mild, and there was no radiographic evidence of erosive joint disease. In contrast to established rheumatoid arthritis, the full remission of arthritis after anti‐tuberculous medication suggests a reactive inflammatory mechanism. Follow‐up anti‐CCP testing was not performed, which represents a limitation of this report.

The lack of clinical response to methotrexate and hydroxychloroquine further argued against active RA and prompted reconsideration of the diagnosis. Interestingly, no TB screening was done before starting methotrexate treatment. It is common practice to screen for latent or active TB before beginning immunosuppressive medication in an area where tuberculosis is endemic. This omission, which is a diagnostic error, highlights the importance of conducting an appropriate infection screening before beginning DMARDs. In hindsight, migratory arthritis and constitutional symptoms, including weight loss, low‐grade fever, and night sweats, ought to have sparked suspicion of an infectious etiology sooner.

This presentation is similar to that reported by Sasaki et al., who described Poncet's disease with high titers of rheumatoid factor and anti‐citrullinated peptide antibodies mimicking rheumatoid arthritis [5]. However, unlike typical RA, our patient had a chest x‐ray which showed consolidation in the right upper lobe, prompting further investigation, which confirmed TB with bronchoalveolar lavage culture, despite a negative IGRA, unlike a previous study, which reported a Patient with migratory arthritis due to TB and positive IGRA [6]. The fibrotic honeycombing changes were interpreted as incidental background lung disease and were not considered contributory to the acute presentation, which was explained by active cavitary tuberculosis. IGRA can be negative in the presence of TB in certain conditions, such as immunocompromised patients, HIV coinfection, central nervous system TB, advanced age, emaciation, and HLA genotype [7]. However, our patient is immunocompetent, and this reflects that TB arthritis is another important cause of false negatives IGRA. The false‐negative IGRA result could have been due to the patient's cumulative exposure to immunosuppressive therapy, particularly systemic corticosteroids. In the clinical setting of probable tuberculosis, IGRA data should be taken cautiously because advanced age and severe weight loss or cachexia may also have decreased the interferon‐gamma response. Unfortunately, diagnosis of TB arthritis can be difficult because of the subtlety of the disease and vague symptoms, especially when there are minimal or latent pulmonary symptoms, as highlighted in prior literature [8, 9]. A recent study done in Japan by Hirokazu Sasaki showed the importance of suspicion of TB in patients who present with RA‐like symptoms or findings [5]. Similarly, another study emphasized the importance of considering TB in patients who present with arthritis with no clear cause, especially in endemic regions [6, 10].

TB can involve the musculoskeletal system in two ways: either direct septic arthritis resulting from hematogenous spread of *Mycobacterium tuberculosis* to bones or joints, leading to tissue destruction, or reactive sterile arthritis (Poncet's disease), a non‐destructive polyarthritis caused by immune‐mediated mechanisms [11, 12]. The presentation in our case was more consistent with reactive tuberculous arthritis (Poncet's disease) because of its non‐destructive, migratory pattern, lack of erosive changes, and rapid recovery following anti‐tuberculous treatment. However, since microbiological confirmation from the affected joint was not obtained due to patient refusal of joint aspiration, direct tuberculous septic arthritis cannot be completely ruled out. The classic example of destructive musculoskeletal tuberculosis is Pott's disease, which involves the vertebrae and adjacent joints. Similar to our case, Ariza et al. reported successful resolution of arthritis with anti‐tuberculous treatment in patients with Poncet's disease [11]. Finally, the resolution of the arthritis with anti‐TB therapy suggests the reactive nature of the arthritis in response to the underlying TB infection.

While most cases of Poncet's disease present simultaneous polyarthritis, our case reports a distinct migratory pattern of joint involvement. This presentation is rare, with only a few prior studies describing TB arthritis as migratory [6, 12, 13]. Additional studies will be required to clarify the features of this rare disease, as ultimately discussed by Wilkinson et al., when they noted they struggled to diagnose their patient as having Poncet's disease [10].

In conclusion, TB should be considered in the differential diagnosis for migratory arthritis, especially in endemic parts of the world and among patients with known risk factors. Early diagnosis of TB and initiation of treatment with anti‐TB therapy will allow for complete resolution of symptoms and prevent complications of the infection. Clinicians should maintain a high index of suspicion as the use of DMARDs in such patients may lead to dissemination of infection even more [5, 10].

## Author Contributions

**Farah Jaafar Mahdi:** conceptualization, project administration, validation. **Mariam Salem Othman:** methodology, resources. **Ahmed Dheyaa Al‐Obaidi:** data curation, writing – original draft. **Mustafa Almusawi:** investigation, validation. **Marafi Jammaa Ahmed:** conceptualization, writing – review and editing. **Aya Ahmed Shimal:** data curation, supervision. **Elian Khalafalla Awadalla:** data curation, visualization. **Marwah Algodi:** conceptualization, writing – original draft.

## Funding

The authors have nothing to report.

## Ethics Statement

Our institution does not require ethical approval for reporting individual cases or case series.

## Consent

Written informed consent was obtained from the patient for publication of this case report and accompanying images. The patient provided consent according to the journal's consent requirements.

## Conflicts of Interest

The authors declare no conflicts of interest.

## Data Availability

Data available on request from the authors.
